# The bidirectional enigma of glaucoma and anxiety: from neuroinflammatory mechanisms to mind-body integrated therapies

**DOI:** 10.3389/fpsyt.2025.1679419

**Published:** 2025-10-29

**Authors:** Bin Lin, Jing Tang, Wei Liang, Dong-kan Li

**Affiliations:** ^1^ Xiamen Eye Center and Eye Institute of Xiamen University, Xiamen, China; ^2^ Xiamen Clinical Research Center for Eye Diseases, Xiamen, Fujian, China; ^3^ Xiamen Key Laboratory of Ophthalmology, Xiamen, Fujian, China; ^4^ Fujian Key Laboratory of Corneal & Ocular Surface Diseases, Xiamen, Fujian, China; ^5^ Xiamen Key Laboratory of Corneal & Ocular Surface Diseases, Xiamen, Fujian, China; ^6^ Translational Medicine Institute of Xiamen Eye Center of Xiamen University, Xiamen, Fujian, China; ^7^ Xiamen Humanity Rehabilitation Hospital, Xiamen, China

**Keywords:** glaucoma, anxiety, bidirectional mechanism, neuroinflammation, mind-body therapy, treatment adherence

## Abstract

**Introduction:**

Glaucoma, a leading cause of irreversible global blindness, has a bidirectional association with anxiety that worsens both conditions. Anxiety prevalence in glaucoma patients (19.07%–25.71%) is much higher than the general population’s 9.8%, and glaucoma patients have a 4.45-fold higher anxiety risk than healthy controls, underscoring the need to synthesize their interactions and interventions.

**Methods:**

A systematic search of PubMed, Web of Science, and Embase (2010–2025) identified studies on glaucoma-anxiety bidirectional relationships, mechanisms, and interventions. Following PRISMA guidelines, 99 studies were screened, with 14 eligible for synthesis.

**Results:**

Bidirectional pathogenesis involves three pathways: (1) Anxiety accelerates glaucoma via hypothalamic-pituitary-adrenal axis overactivation (43% lower retinal ganglion cell [RGC] survival), sympathetic catecholamine release, and microglial IL-1β/TNF-α secretion (P<0.001); (2) Glaucoma induces anxiety through RGC apoptosis-driven HMGB1/TLR4/NF-κB activation and abnormal amygdalar connectivity; (3) Anxiety correlates with 40% reduced treatment adherence, faster visual field progression (-1.5 dB/year), and 30% higher postoperative complications. Gaps include limited longitudinal data and unstandardized psychological interventions.

**Discussion:**

“Mind-body integrated therapy” is prioritized: cognitive-behavioral therapy boosts adherence by 76%, biofeedback lowers intraocular pressure by 4.8 mmHg, and SSRIs alleviate anxiety safely. Future research should focus on biomarker-guided and anti-inflammatory interventions to shift management toward physiological-psychological co-care.

## 1 Introduction: the interdisciplinary significance of glaucoma and anxiety​

Glaucoma, a leading cause of irreversible blindness worldwide, imposes a significant mental health burden on patients. A meta-analysis of 16 cross-sectional studies (including glaucoma patients) reported a pooled anxiety prevalence of 19.07% (95% CI: 13.34%–26.51%) among this population, while a study of 210 Brazilian glaucoma patients specifically documented a higher anxiety prevalence of 25.71%–both substantially higher than the 9.8% anxiety prevalence in the general population (WHO data) (1–3). Case-control studies further confirm that glaucoma patients face a 4.45-fold increased risk of anxiety compared to healthy controls (1, 3), with anxiety severity positively correlated with disease progression (2).

A bidirectional mechanism links glaucoma and psychological states. Physiologically, progressive vision loss, complex medication regimens, and fear of visual deterioration form the basis of psychological vulnerability (4, 5). Psychologically, anxiety reduces treatment adherence by approximately 40%, accelerating disease progression and creating a “physiological-psychological vicious cycle” (6, 7).

Mental health issues exert multidimensional impacts on glaucoma management. Psychological comorbidities significantly disrupt medication adherence (3, 4) and accelerate visual field defect progression (6). Additionally, anxiety induces multidimensional functional impairments, underscoring the need for collaborative ophthalmological-psychiatric interventions (5).

Given the significant gaps in longitudinal causal evidence, genetic validation, and psychological intervention strategies, a systematic review of the relationship between glaucoma and anxiety is urgently needed. By integrating fragmented cross-sectional data, dissecting bidirectional mechanisms, and synthesizing intervention approaches, this review aims to bridge research gaps and inform future longitudinal cohort studies and large-scale genetic explorations. Clinically, it seeks to provide ophthalmologists with a standardized framework for identifying psychological comorbidities, facilitating the development of personalized protocols that integrate psychological interventions with pharmacological therapy. These efforts aim to enhance treatment adherence, decelerate disease progression, and ultimately improve visual function and quality of life in glaucoma patients while promoting the optimization of interdisciplinary diagnostic and therapeutic models.

## 2 Methods

### 2.1 Literature search strategy

**Figure 1:**
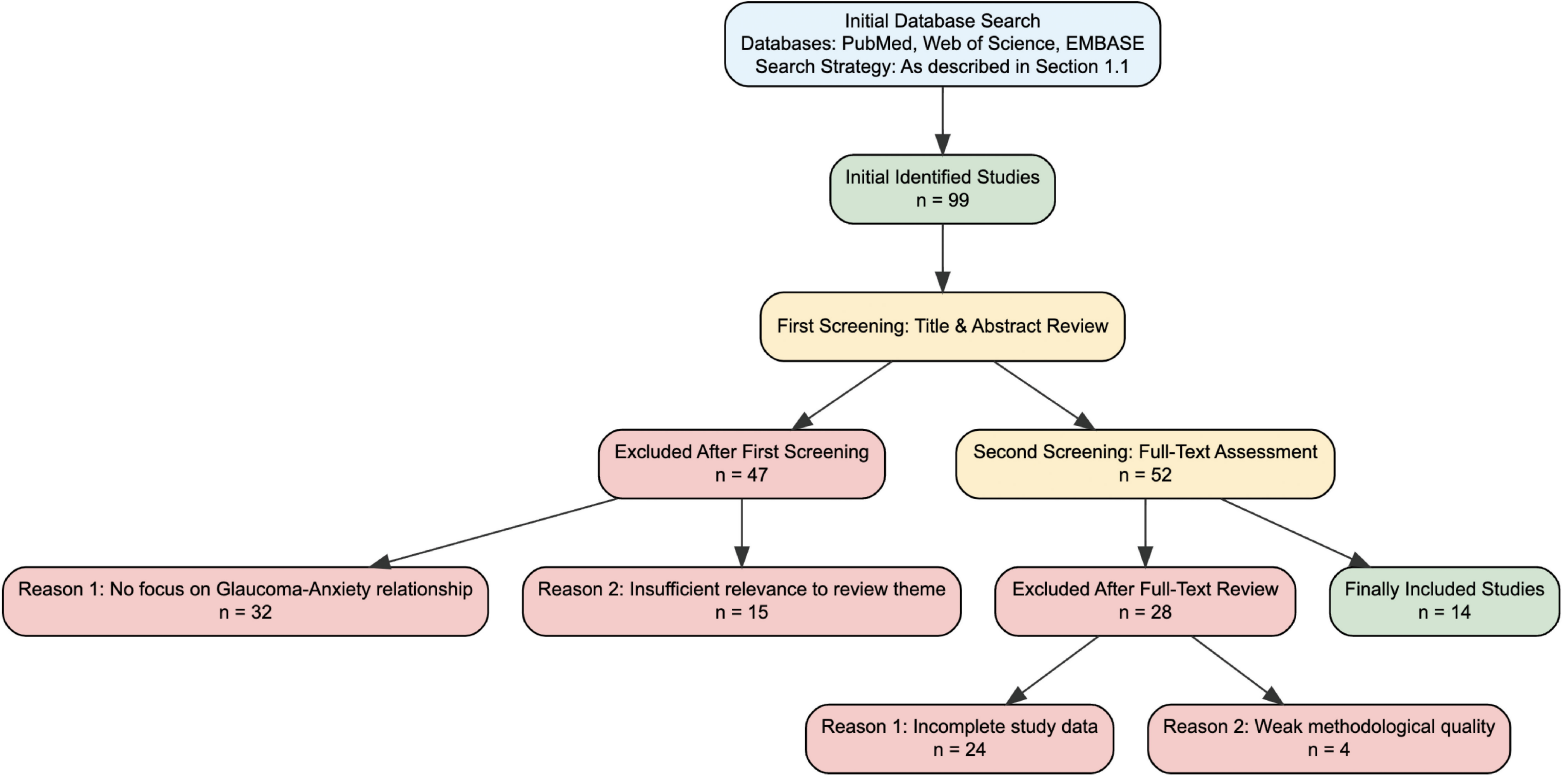
Flow diagram of literature screening process. A total of 99 references were initially identified via the study’s search strategy. After screening, 75 individuals were excluded: 32 did not focus on the glaucoma-anxiety relationship, 24 had incomplete data, 15 had low topic relevance, and 4 had weak methodological quality. Finally, 14 studies meeting all criteria were included, following PRISMA guidelines.

A systematic literature search was performed in PubMed, Web of Science, and Embase to identify relevant studies published between January 2010 and August 2025. The search strategy was designed to capture the bidirectional relationship between glaucoma and anxiety, their underlying mechanisms, and associated interventions, aligning with the core focus of this review. The specific Retrieval Strategies and literature inclusion/exclusion criteria are as follows. The detailed literature screening flowchart is presented in **Figure 1**.

The final combined Boolean search string was constructed as follows:

(“glaucoma” OR “open-angle glaucoma” OR “angle-closure glaucoma” OR “primary open-angle glaucoma” OR “retinal ganglion cell damage”) AND (“anxiety” OR “anxiety disorders” OR “psychological stress” OR “emotional distress” OR “neuroticism”) AND (mechanism OR pathogenesis OR “neuroinflammation” OR “neuroendocrine” OR “treatment adherence” OR “cognitive-behavioral therapy” OR “SSRIs” OR “mind-body therapy” OR “TNF-alpha” OR “IL-6” OR “BDNF”) AND (“2010/01/01”[PDAT]: “2025/06/01”[PDAT])

To comprehensively synthesize the latest advancements in the bidirectional relationship between glaucoma and anxiety, we employed a hybrid approach combining systematic literature retrieval with flexible expansion to ensure rigor and innovation. These captures established evidence on epidemiological associations, neuroinflammatory mechanisms, and emerging insights into their dynamic interplay, bridging foundational knowledge with cutting-edge developments.

### 2.2 Inclusion criteria

Study type: Original research (cohort studies, case-control studies, randomized controlled trials), systematic reviews, meta-analyses, and preclinical studies (animal models, *in vitro* experiments) focusing on the glaucoma-anxiety relationship.

Content: Studies investigating comorbidity, bidirectional associations, or causal links between glaucoma (any subtype) and anxiety; mechanistic studies addressing neuroinflammation, neuroendocrine dysfunction, or vascular regulation; and intervention studies evaluating psychological, pharmacological, or integrated therapies for co-occurring glaucoma and anxiety.

Data availability: Studies providing extractable data on epidemiological metrics (prevalence, odds ratios), mechanistic markers, or clinical outcomes (treatment adherence, intraocular pressure changes).

### 2.3 Exclusion criteria

Studies focusing solely on glaucoma or anxiety without addressing their comorbidity.

Case reports, letters to the editor, or narrative reviews lacking original data or rigorous analysis.

Studies investigating mechanisms unrelated to neuroinflammation, neuroendocrine pathways, or psychological-behavioral factors.

Duplicate publications (priority given to the most recent or largest study).

Non-English articles or studies with insufficient data for synthesis.

## 3 Epidemiology: the comorbid pattern of glaucoma and anxiety

### 3.1 The high prevalence of anxiety in glaucoma patients

Glaucoma imposes a significant mental health burden on patients, with anxiety being notably more prevalent among this group than in the general population. Case-control studies indicate that glaucoma patients have a 4.45-fold increased risk of anxiety compared to controls (3), and a meta-analysis confirms an odds ratio (OR) of 2.11 (95% CI 1.22–3.66) (8).

Risk stratification highlights multiple influencing factors (9). In terms of the glaucoma subtype, angle-closure glaucoma patients exhibit a significantly higher anxiety risk than open-angle patients, with acute angle-closure crisis patients more prone to acute anxiety due to sudden vision loss (5). Demographically, female patients have a 1.99-fold higher anxiety risk than males (10, 11), while those with lower education levels show a 2.3-fold increased comorbidity rate (5), and individuals of lower socioeconomic status face elevated risk (8). Clinically, patients with advanced visual field defects (MD >12 dB) have a 3.5-fold higher anxiety risk (2, 5), surgical candidates show 1.41-fold higher anxiety scores than those on medical therapy (12), and patients with ≥3 daily medication administrations have a 2.8-fold increased risk (4). Regarding comorbidities, a history of substance abuse elevates risk by 4.2-fold especially nicotine (13, 14).

This high prevalence is explained by multiple mechanisms. Vision-related functional impairment restricts daily activities, treatment complexity such as difficulty with eyedrop administration induces therapy-related anxiety, and fear of blindness creates persistent psychological stress (4, 5). Neurobiologically, this may involve concurrent degeneration of retinal ganglion cells and limbic system structures (15).

### 3.2 Anxiety and its associations with glaucoma onset: from emotional stress to medication-related triggers

Epidemiological studies indicate a significant comorbid relationship between glaucoma and anxiety. Multiple studies highlight that emotional stress plays a pivotal role in acute angle-closure glaucoma attacks (16), with approximately 65–75% of acute episodes associated with intense emotional fluctuations such as anger and panic (17, 18). This association may stem from autonomic nervous system disorders induced by emotional stress, leading to pupil dilation, angle closure, and abrupt intraocular pressure elevation (19, 20). Notably, while drug-induced acute angle-closure glaucoma is relatively rare, oral administration of anticholinergic-containing medications in some anxiety patients may trigger acute episodes through similar mechanisms (21). On the other hand, long-term anxiety may promote glaucoma progression by affecting autonomic regulation, inflammatory cytokine levels, and different pathways (22, 23).

## 4 Bidirectional pathogenesis: imbalance of neuroendocrine-immune network

### 4.1 Pathological pathways of anxiety-induced glaucoma

Anxiety influences glaucoma pathogenesis through the first primary pathways: neuroendocrine and vascular regulation. In the sympathetic nervous pathway, chronic anxiety triggers sustained catecholamine release, which not only causes acute intraocular pressure elevation via mechanical blockage of the anterior chamber angle by the iris root (24) but also induces structural changes in the aqueous outflow pathway through α1-adrenergic receptor-mediated trabecular meshwork cell contraction and extracellular matrix remodeling (25, 26). Concomitantly, vasoconstriction-induced microcirculatory dysfunction in the optic nerve head exacerbates ischemic injury to retinal ganglion cells (RGCs), with experimental data showing a significantly higher RGC apoptosis rate (P<0.001) in glaucoma animal models (27), closely associated with Matrix Metalloproteinase-9 (MMP-9)-mediated extracellular matrix degradation and mitochondrial dysfunction (25, 28).

Dysregulation of the hypothalamic-pituitary-adrenal (HPA) axis constitutes the second key pathway. Prolonged anxiety elevates glucocorticoid levels, affecting glaucoma progression through dual mechanisms: cortisol directly inhibits trabecular meshwork cell phagocytosis by activating glucocorticoid receptors, leading to abnormal protein deposition in the aqueous outflow pathway (29); HPA axis dysfunction downregulates retinal neurotrophic factors such as brain-derived neurotrophic factor (BDNF), as validated in the chronic unpredictable mild stress (CUMS) animal model, where RGC survival rate decreases by 43% (30). Glucocorticoids promote trabecular meshwork fibrosis by upregulating Transforming Growth Factor-beta (TGF-β) signaling, a mechanism confirmed in aqueous humor samples from primary open-angle glaucoma (POAG) patients (31).

Additionally, activation of neuroinflammatory and cell death pathways may form a third route linking anxiety to glaucoma. Chronic psychological stress prompts microglia to release proinflammatory cytokines such as Interleukin-1 beta (IL-1β) and Tumor Necrosis Factor-alpha (TNF-α), which not only exacerbate optic nerve inflammation via the Nuclear Factor-kappa B (NF-κB) pathway (29) but also accelerate RGC death by regulating ferroptosis-related protein Glutathione Peroxidase 4 (GPX4) expression (32). Clinical studies show that aqueous humor 8-hydroxydeoxyguanosine (8-OHdG) levels in anxious glaucoma patients are 2.3-fold higher than controls, indicating cumulative oxidative stress damage (33). Finally, anxiety-related white matter microstructural changes may disrupt functional connectivity between the visual cortex and limbic system, forming an “anxiety-neurodegeneration” vicious cycle (15). Notably, microglia-mediated inflammation is also recognized as one of the primary pathological mechanisms of anxiety disorders. Studies have found that abnormal activation of microglia (e.g., via the Warburg effect or Pyruvate Kinase M2/Hypoxia-Inducible Factor-1 alpha (PKM2/HIF-1α) pathway) can lead to synaptic dysfunction and anxiety-like behaviors (34). This research indicated that in anxiety models, excessive activation of microglia is associated with impaired hippocampal synaptic function, while regulating microglial metabolism can alleviate anxiety behaviors. Furthermore, activation of microglia in specific brain regions (such as the amygdala) can directly induce anxiety (35, 36).

### 4.2 Pathways of glaucoma-induced anxiety

The psychophysiological pathways between glaucoma and anxiety involve multi-level complex mechanisms. From the perspective of visual function impairment, progressive visual field loss triggers profound fear of vision loss in patients, with this “blindness anxiety” escalating as the disease advances. Studies show that the prevalence of anxiety in glaucoma patients reaches 19.07%, significantly higher than that in the general population, and correlates with disease severity (1, 2). This psychological burden stems not only from fear of blindness but also from social function impairment—for example, reduced driving and reading abilities lead to social isolation, further increasing anxiety risk (4, 37).

**Figure 2:**
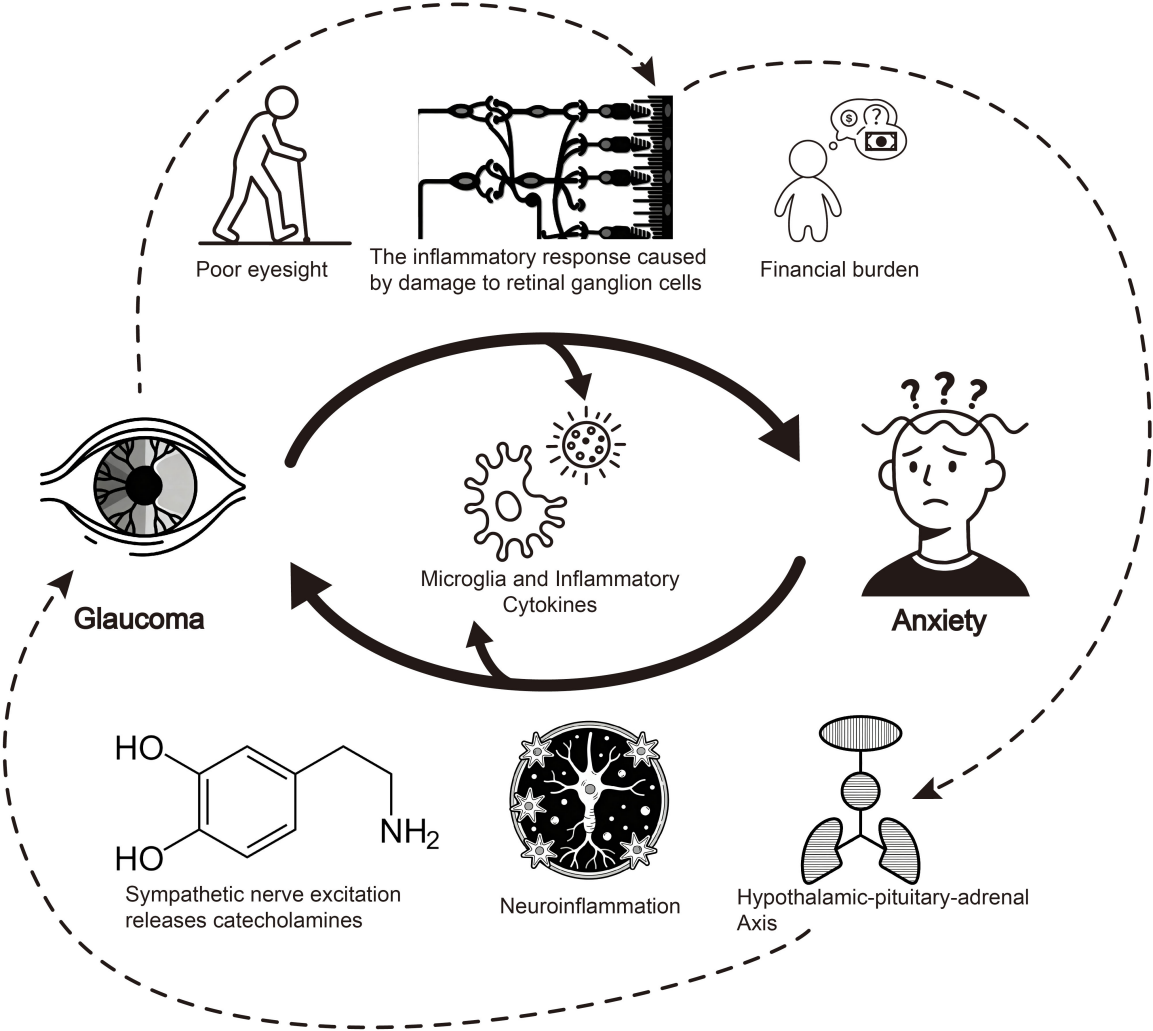
Schematic diagram of bidirectional pathways between glaucoma and anxiety. This diagram illustrates the bidirectional mechanisms linking glaucoma and anxiety. Glaucoma induces anxiety via poor eyesight, retinal ganglion cell damage-triggered inflammation, and financial burden. Conversely, anxiety promotes glaucoma progression through neuroinflammation, sympathetic nerve-mediated catecholamine release, and hypothalamic-pituitary-adrenal axis dysregulation. Among these, the inflammatory cascade acts as a critical bridge—neuroinflammation exacerbates optic nerve injury primarily through microglial activation and the release of proinflammatory factors (e.g., TNF-α, IL-1β); furthermore, this inflammation may cross the blood-brain barrier to disrupt limbic system function, with particular impacts on emotion-regulating centers such as the amygdala, thereby contributing to anxiety development. Notably, as indicated by the dashed arrows in the figure, HPA axis dysfunction leads to increased glucocorticoid levels, which not only impair the phagocytic function of the trabecular meshwork but also promote trabecular meshwork fibrosis, collectively accelerating glaucoma progression and further optic nerve damage and atrophy. Additionally, proinflammatory factors (e.g., TNF-α) involved in this pathological process may form a positive feedback loop by further exacerbating HPA axis dysregulation, reinforcing the bidirectional vicious cycle between glaucoma and anxiety.

At the neurobiological level, glaucoma influences emotional regulation through neuroinflammatory cascades. Apoptosis of retinal ganglion cells (RGCs) releases high-mobility group box 1 (HMGB1), activating microglia and promoting the release of proinflammatory cytokines TNF-α and IL-1β (38, 39). These inflammatory mediators cross the blood-brain barrier to affect limbic system function, particularly emotion-regulating centers like the amygdala (40, 41). Research indicates that activation of High-Mobility Group Box 1/Toll-Like Receptor 4/Nuclear Factor-kappa B (HMGB1/TLR4/NF-κB) and Tumor Necrosis Factor-alpha/Tumor Necrosis Factor Receptor 1/Nuclear Factor-kappa B (TNF-α/TNFR1/NF-κB) signaling pathways plays a key role in neuroinflammation and anxiety-like behaviors (39, 42). Notably, proinflammatory factors such as TNF-α—released during this neuroinflammatory cascade—may further disrupt HPA axis homeostasis, creating a positive feedback loop that not only exacerbates neuroinflammation but also accelerates glaucomatous optic nerve damage (43). Additionally, resting-state Functional Magnetic Resonance Imaging (fMRI) in glaucoma patients reveals abnormal amygdala functional connectivity, which may serve as the neural basis for emotional regulation disorders (44). These neurobiological and psychophysiological cascades collectively underpin how glaucoma and anxiety interact with each other, and the interconnections between these pathways are visually synthesized in **Figure 2**.

Notably, the link between retinal pathological changes (e.g., RGC apoptosis) and anxiety remains to be fully validated by direct experimental evidence, though indirect mechanistic clues and analogous pathways provide supportive insights. Retinal ganglion cells (RGCs), particularly intrinsically photosensitive RGCs (ipRGCs), have been confirmed to regulate emotional behaviors via the retinal-brain axis-for instance, short-term exposure to intense light activates ipRGCs and triggers anxiety-like behaviors in rodents, likely through signaling to hypothalamic and limbic structures (45). While this pathway does not explicitly involve Tumor Necrosis Factor-alpha (TNF-α), prior studies have established that TNF-α is a key proinflammatory cytokine released during RGC apoptosis, mediating retinal neuroinflammation and exacerbating optic nerve damage (46). Given the anatomical and functional connectivity of the retina to emotion-regulating brain regions, it is plausible that TNF-α released from apoptotic RGCs may propagate signals through similar retinal-hypothalamic-limbic pathways to modulate anxiety. However, direct evidence-such as *in vivo* observations of anxiety-like behaviors induced by retinal TNF-α overexpression or neutralization in animal models of glaucoma—remains lacking, highlighting a critical direction for future research to confirm this bidirectional pathological link.

To synthesize the complex interplay, **Table 1** summarizes the key bidirectional mechanisms linking glaucoma and anxiety, including neuroendocrine dysregulation, neuroinflammatory cascades, and structural brain changes, with supporting references.

**Table 1:** Bidirectional mechanisms between glaucoma and anxiety.

Direction of interaction	Mechanism category	Specific mechanisms	References
Anxiety → Glaucoma	Neuroendocrine Pathway	Chronic anxiety elevates glucocorticoids, inhibiting trabecular meshwork phagocytosis and downregulating BDNF.	(30, 31)
Anxiety → Glaucoma	Sympathetic Activation	Sustained catecholamine release causes trabecular meshwork contraction and optic nerve head microcirculatory dysfunction.	(25, 26)
Anxiety → Glaucoma	Neuroinflammation	Microglia release IL-1β and TNF-α, exacerbating optic nerve inflammation via NF-κB pathway.	(33, 37)
Glaucoma → Anxiety	Neuroinflammatory Cascade	RGC apoptosis releases HMGB1, activating HMGB1/TLR4/NF-κB and TNF-α/TNFR1/NF-κB pathways affecting limbic system.	(38, 41)
Glaucoma → Anxiety	Structural Brain Changes	Abnormal amygdala functional connectivity observed via resting-state fMRI in glaucoma patients.	(42)
Bidirectional	Oxidative Stress	Increased aqueous humor 8-OHdG levels in anxious glaucoma patients indicate cumulative oxidative damage.	(34)

BDNF, Brain-Derived Neurotrophic Factor; IL-1β, Interleukin-1 beta; TNF-α, Tumor Necrosis Factor-alpha; NF-κB, Nuclear Factor-kappa B; RGC, Retinal Ganglion Cell; HMGB1, High-Mobility Group Box 1; TLR4, Toll-Like Receptor 4; TNFR1, Tumor Necrosis Factor Receptor 1; fMRI, Functional Magnetic Resonance Imaging; 8-OHdG, 8-hydroxydeoxyguanosine.

## 5 Impact of anxiety on glaucoma clinical outcomes

### 5.1 Reduced treatment adherence

Studies demonstrate a significant negative correlation between anxiety symptoms and treatment adherence in glaucoma patients. Multiple cross-sectional studies show that during the Coronavirus Disease 2019 (COVID-19) pandemic, anxiety severity directly correlated with reduced treatment adherence, indirectly compromising therapeutic efficacy. Specifically, assessments using the Beck Anxiety Inventory (BAI) revealed that patients with severe anxiety were more prone to missed eyedrop administrations and had significantly higher non-attendance rates for follow-up visits than non-anxious patients (47). Notably, a study involving 111 glaucoma patients found that depressive symptoms—often comorbid with anxiety—increased the risk of low treatment adherence by 38-fold (48).

Mechanistically, anxiety and poor glaucoma outcomes form a vicious cycle. Anxiety not only directly affects patients’ medication compliance and follow-up attendance but may also exacerbate glaucoma progression via neurobiological pathways (4, 6). Research confirms that anxiety symptoms correlate significantly with negative visual function metrics such as mean deviation (MD), indicating that anxiety may accelerate glaucoma progression (47). Concurrently, deteriorating visual function further intensifies patient anxiety, creating an unbroken pathological cycle (49, 50). This bidirectional relationship was particularly pronounced during the pandemic when glaucoma patients exhibited a non-adherence rate as high as 28.18% (51).

Clinical practice requires special attention to the multiple impacts of anxiety on glaucoma management. Evidence shows that integrating mental health interventions can significantly improve patient outcomes (7). Strategies such as enhancing physician-patient relationship quality and implementing medication reminder technologies effectively alleviate anxiety and improve adherence (52–54). Additionally, healthcare providers should prioritize female patients and those with multiple comorbidities, as these groups face a 5.25-fold increased risk of anxiety comorbidity (55). These findings underscore the necessity of incorporating psychological assessment and intervention into routine glaucoma care (1, 56).

### 5.2 Accelerated disease progression​

Studies have demonstrated a significant association between anxiety and accelerated glaucoma progression. Patients with high anxiety exhibit faster rates of visual field defect progression (-1.5 dB/year) and retinal nerve fiber layer thinning (-4.2 μm/year), nearly twice the rates observed in low-anxiety groups (-0.8 dB/year and -2.3 μm/year, respectively) (57). This discrepancy may stem from the impact of anxiety on intraocular pressure fluctuations and optic nerve microcirculation, as high-anxiety patients are more prone to optic disc hemorrhage (28% vs. 9%), a confirmed risk factor for glaucoma progression (58). Research also shows a positive correlation between anxiety levels and the frequency of optic disc hemorrhage, which may accelerate retinal nerve fiber layer damage by affecting microvascular function in the optic nerve head (59).

Pathophysiologically, anxiety influences glaucoma progression through multiple pathways. Chronic psychological stress leads to overactivation of the sympathetic nervous system, causing intraocular pressure fluctuations and abnormal regulation of optic nerve blood flow (24). A longitudinal study revealed that anxious patients exhibit more pronounced decreases in optic nerve head vascular density, with this microcirculatory dysfunction directly correlating with the rate of retinal nerve fiber layer thinning (60). Additionally, anxiety may indirectly exacerbate disease progression by impairing treatment adherence, as high-anxiety patients are more likely to engage in non-compliant medication use, forming a vicious cycle of disease progression and psychological distress (6).

Clinical observations further confirm the close association between anxiety and structural/functional damage in glaucoma. Optic disc hemorrhage, a key marker of glaucoma progression, occurs more frequently in high-anxiety patients, often at the margins of retinal nerve fiber layer defects (58). This hemorrhagic pattern suggests that anxiety exacerbates the interaction between mechanical injury and vascular dysfunction in the optic nerve head (61). Notably, the impact of anxiety on glaucoma progression may have a cumulative effect: as disease duration increases, psychological factors progressively intensify negative effects on visual function and quality of life (50). These findings underscore the necessity of integrating psychological assessment and intervention into glaucoma management (56).

### 5.3 Deteriorated surgical outcomes

Anxiety in glaucoma patients is significantly associated with compromised surgical prognosis. Patients with comorbid anxiety often exhibit delayed intraocular pressure recovery after surgery, potentially linked to abnormal activation of the neuroendocrine system under stress (24, 37). Multiple clinical datasets show that these patients face a 30% increased risk of postoperative complications, including anterior chamber hemorrhage and drainage tube obstruction (62, 63). This association stems from two mechanisms: firstly, anxiety-induced elevation in cortisol levels may impair wound healing; secondly, reduced treatment adherence in anxious patients compromises the regularity of postoperative medication and timeliness of follow-ups (6, 7). Notably, female patients and those with systemic comorbidities exhibit higher anxiety-related surgical risks, with odds ratios (OR) of 5.25 and 2.82, respectively (2, 55). Clinical observations confirm that the negative impact of anxiety on surgical outcomes intensifies with longer disease duration and more severe visual impairment (5, 50). This highlights the need for clinicians to integrate psychological assessment into perioperative management, leveraging interdisciplinary collaboration to improve patients’ physical and mental states (4).

## 6 Clinical management strategies: from “intraocular pressure reduction” to “mind-body integrated therapy”​​

### 6.1 Application of anxiety screening tools​

To optimize the clinical utility of these screening tools in mind-body integrated therapy, a standardized two-step implementation workflow is recommended to ensure consistency and efficiency in clinical practice:

First, initial screening should be integrated into key clinical touchpoints for all glaucoma patients, including the first diagnosis visit, pre-surgical assessment, and 1-month post-surgical follow-up. The GAD-7 scale is selected as the primary tool here—its short completion time minimizes disruption to ophthalmic consultation workflows, and its strong reliability in the glaucoma population (Cronbach’s α=0.943) ensures accurate identification of potential anxiety (64). A GAD-7 score of ≥8 is set as the threshold to trigger further evaluation, as this cut-off aligns with clinical validation for detecting relevant anxiety symptoms in ophthalmic settings, consistent with the utility of these scales in prior glaucoma-related mental health assessments (56).

Second, confirmatory evaluation is essential for patients with GAD-7 ≥8: the PHQ-9 scale should be administered concurrently to rule out comorbid depressive symptoms, which often coexist with anxiety and independently impact treatment adherence (56). For patients with PHQ-9 scores ≥10, a structured handoff to psychiatric care is necessary to confirm diagnoses and initiate targeted psychological intervention, ensuring alignment with the holistic goals of mind-body integrated therapy—an approach supported by evidence linking psychological comorbidity assessment to improved glaucoma management outcomes.

Notably, screening frequency should be tailored to disease severity to allocate resources effectively: patients with advanced glaucoma (characterized by mean deviation [MD] >12 dB) or recent surgical intervention (within 6 months) require semi-annual screening, given their higher psychological vulnerability (5). In contrast, stable early-stage glaucoma patients (MD ≤6 dB) with consistent intraocular pressure control may undergo annual screening, as their risk of anxiety onset or exacerbation is relatively lower (51). This stratified approach ensures that mind-body integrated care is both accessible and resource-efficient, reflecting prior findings on the association between glaucoma severity, treatment stage, and psychological distress.

### 6.2 Core techniques of psychological intervention​

In psychological interventions for glaucoma, cognitive-behavioral therapy (CBT) significantly improves treatment adherence and psychological adaptability by helping patients restructure catastrophic cognitions about blindness, with studies showing a 76% effectiveness rate (7, 65). Biofeedback therapy, through real-time monitoring of electromyographic/electrodermal signals, trains patients to autonomously regulate sympathetic nerve activity, with clinical data demonstrating a 4.8 mmHg reduction in intraocular pressure (66, 67). This physiological-psychological interactive regulation mechanism offers new insights for glaucoma treatment. Music therapy combined with relaxation training—particularly theta-wave music—reduces intraocular pressure by 10–15% through modulating limbic system activity, potentially via music-induced parasympathetic activation and cortisol level reduction.

These interventions collectively form an integrated “mind-body integrated therapy” model. Meditation (30–60 minutes daily) and autogenic training have also been proven to improve intraocular pressure and optic nerve blood flow (7, 68), while motivational interviewing and psychological care enhance long-term efficacy by strengthening self-management capabilities (69). Notably, these non-pharmacological interventions, as supplements to conventional treatments, are particularly suitable for glaucoma patients with comorbid anxiety (37). Their core lies in achieving synergistic effects of intraocular pressure control and psychological symptom relief through multi-target regulation of neuroendocrine-immune-vascular pathways (50, 70). The early visual rehabilitation combined with psychological counseling proposed in the UK can specifically address the association between visual impairment and anxiety, being particularly suitable for patients with low socioeconomic status (71). The “Eye Yoga” trial in India has confirmed that mind-body training can improve intraocular pressure and retinal blood flow (72), and it requires no complex equipment-only training for ophthalmic nurses is needed for implementation. While psychological counseling under this model demands support from professionals, low-cost interventions like “Eye Yoga” are applicable in resource-limited settings. Moreover, practices such as meditation align with the cultural perceptions of some Asian populations, and their acceptance can be enhanced through localized adjustments to the training format.

The effectiveness of mind-body integrated therapy in clinical settings relies on optimized multidisciplinary collaboration (MDC) models. Key improvements to such models include establishing dedicated coordination mechanisms within clinical teams to facilitate communication between ophthalmological and psychological care providers, ensuring timely referral of patients with anxiety-related needs, and consistent tracking of intervention progress. Additionally, regular interdisciplinary case discussions can be conducted to align biological treatment goals (e.g., intraocular pressure control) with psychological intervention plans, adapting strategies based on patients’ comprehensive clinical status. Remote collaboration tools may also be leveraged to support continuous care for patients with limited accessibility, enabling shared access to clinical data and dynamic adjustment of integrated therapy protocols. Incorporating periodic outcome assessments—focused on anxiety symptom changes, treatment adherence, and glaucoma-related clinical indicators—will further refine the MDC model, ensuring it effectively supports the delivery of patient-centered mind-body integrated care.

For example, the German collaborative stepped care model conducts hierarchical interventions relying on a multidisciplinary team (ophthalmologists, psychiatrists, general practitioners). With standardized assessment tools (73), it can significantly improve patients’ quality of life within 0–6 months and maintain long-term effects (74, 75), which can be easily integrated into the ophthalmic diagnosis and treatment process of institutions with established multidisciplinary teams. However, its implementation requires sufficient specialized human resources and considerable time investment. In the current implementation across many countries, it is still necessary to coordinate the differences in work modes among different departments to adapt to clinical practice.

In view of this situation, a randomized controlled trial in Denmark showed that the collaborative care model is more effective in reducing anxiety symptoms compared with traditional consultation (75). Studies in Latin America have proposed a standardized process for integrating mental health services into primary care and coordinating resources based on case management manuals (76), which can be easily replicated in primary care settings. Its implementation requires the establishment of a referral network between primary care and specialist departments; while the cost of material development is low, training for primary care personnel is necessary. A prerequisite for its successful implementation is to address the issue of weak mental health service capabilities at the primary care level to ensure intervention effectiveness.

### 6.3 Pharmacological combination therapy​

In the comprehensive management of glaucoma patients, the selection of anxiolytic medications must balance intraocular pressure safety and psychological symptom relief. Selective serotonin reuptake inhibitors (SSRIs) such as sertraline and escitalopram serve as first-line recommendations, as they exert no significant effect on pupil diameter and existing evidence shows no substantial association between these agents and glaucoma risk (77). Notably, escitalopram demonstrates clear anxiolytic effects in animal studies (78), while sertraline—one of the most commonly used SSRIs in clinical practice—is administered more frequently than other medications of the same type (79). Caution is warranted for benzodiazepines, which may increase glaucoma risk by inducing angle closure mechanisms and should be used judiciously (80).

Regarding optimization of glaucoma pharmacotherapy, fixed combination formulations significantly enhance treatment adherence by reducing medication frequency (81, 82). Studies show that users of fixed combination regimens exhibit substantially higher medication adherence than those on non-fixed combination therapies (p<0.001) (83, 84), with this advantage being more pronounced in formulations containing two or three medications (85, 86). Importantly, fixed combination preparations not only improve clinical efficacy and safety but also alleviate the burden of polypharmacy (87), which is particularly crucial for glaucoma patients requiring long-term medication (88, 89).

## 7 Discussion and future directions​

The current debate regarding the relationship between glaucoma and anxiety focuses on determining the causal direction. Molecular mechanism studies reveal that dynamic changes in biomarkers such as BDNF and IL-6 may provide key evidence: salivary BDNF levels in glaucoma patients are specifically correlated with anxiety severity (90), while animal experiments have confirmed that TNF-α and IL-6 inhibitors can improve anxiety symptoms by upregulating hippocampal BDNF expression (91, 92), providing a pathophysiological basis for the role of neuroinflammatory pathways (involving the TNF-α/IL-6/BDNF axis) in the comorbidity of the two (93, 94). The bidirectional mechanisms outlined in **Table 1** provide a structured framework to resolve this debate. For instance, the ‘anxiety→glaucoma’ pathway in **Table 1** highlights HPA axis overactivation as a key driver—cortisol elevation not only inhibits trabecular meshwork phagocytosis but also upregulates TGF-β to promote fibrosis, which synergizes with sympathetic catecholamine release to exacerbate aqueous outflow obstruction. This mechanistic synergy explains why anxious glaucoma patients exhibit higher intraocular pressure fluctuations than non-anxious counterparts. Conversely, **Table 1**’s ‘glaucoma→anxiety’ pathway emphasizes RGC apoptosis-triggered HMGB1/TLR4/NF-κB activation, which aligns with clinical data showing higher anxiety prevalence in advanced glaucoma. The overlap in neuroinflammatory mediators crosses both directions in **Table 1**, further supporting inflammation as a shared pathological hub, justifying anti-inflammatory interventions for comorbid patients.

In terms of technological applications, predictive models integrating glaucoma severity, aqueous humor cytokine profiles (95), and personality scales remain to be established. Beyond such predictive modeling, insights from preclinical and clinical neurobiology also point to promising technical avenues: in anxiety models, mice with high anxiety-like behaviors exhibit increased microglial density in specific brain regions (e.g., amygdala, prefrontal cortex) (36), which may correlate with abnormal brain region activity observed in patients with anxiety disorders via human neuroimaging. Meanwhile, several studies have mentioned that targeting microglia enables early detection of glaucoma progression (96, 97), suggesting that *in vivo* monitoring could potentially be achieved through neuroimaging techniques in the future. Notably, **Table 1** also reveals unmet needs in current technological translations. For example, the ‘microglial activation’ mechanism in both bidirectional pathways (**Table 1**) is well-supported by preclinical data (e.g., PKM2/HIF-1α-mediated microglial dysfunction) but lacks *in vivo* imaging validation in humans. While neuroimaging has identified amygdalar connectivity abnormalities in glaucoma patients-as noted in **Table 1**’s ‘structural brain changes’-it has not yet quantified microglial density in emotion-regulating brain regions (e.g., amygdala, prefrontal cortex) to confirm their role as a ‘bridge’ between retinal injury and anxiety. This gap highlights the need for future neuroimaging studies that target microglial activation.

Complementing these diagnostic technologies, therapeutic-focused technical developments are also underway. Beyond the previously mentioned “early visual rehabilitation combined with psychological counseling” from the UK, “Eye Yoga” from India, and the “collaborative stepped care model” from Germany, additional novel therapeutic advancements have emerged. Remote interventions based on VR exposure therapy, under the guidance of psychologists, can help patients gradually adapt to situations that may trigger anxiety and reduce psychological stress, but this technology also needs further development (98). Additionally, remote psychological guidance delivered via mobile phones or computers offers another flexible approach, allowing patients to learn strategies for adjusting negative disease-related cognitions; compared to traditional anxiety questionnaires, this method also enables earlier identification of patients who conceal emotional problems due to “fear of discrimination” (99).

Future research should also focus on evaluating the dual benefits of anti-inflammatory drugs (100, 101) while exploring the dynamic association between digital phenotypic markers and inflammatory markers under multidisciplinary collaboration models (4, 29). It should be noted that the correlation between aqueous humor IL-6 levels and anxiety severity in existing evidence still needs to be validated in large samples (90, 102), and data on the effects of anti-IL-17/23 drugs on anxiety are still lacking (100, 101). However, it is currently known that psychological interventions can improve patient outcomes by regulating these neuroinflammatory pathways, providing new ideas for the comprehensive treatment of glaucoma (7). These findings emphasize the importance of paying attention to the mental health of patients in glaucoma management and the need to adopt comprehensive biological-psychological-social intervention strategies (50).

To address the identified limitations of insufficient longitudinal data, inadequate genetic validation, and the lack of quantitative data highlighted in this review, future studies should adopt targeted design strategies. During the literature search for this review, a substantial number of studies were found to have insufficient data quantification, which rendered some conclusions less definitive—this underscores the critical importance of prioritizing data quantification in future experimental designs to facilitate subsequent statistical analyses and causal inference calculations. For longitudinal investigations, prospective cohort studies with extended follow-up (e.g., 5–10 years) are recommended, integrating repeated and quantitatively standardized assessments of glaucoma progression (via visual field testing with precise mean deviation values, retinal nerve fiber layer imaging with thickness measurements), anxiety severity (using validated scales like GAD-7/PHQ-9 with scoring thresholds for severity stratification), and neuroinflammatory biomarkers (e.g., aqueous humor IL-6, serum BDNF with exact concentration measurements). Such designs will enable robust quantification of bidirectional temporal relationships—for instance, whether baseline anxiety scores predict subsequent annual rates of glaucoma progression, or vice versa. For genetic validation, genome-wide association studies (GWAS) with larger sample sizes (encompassing diverse ethnicities) should be conducted to identify shared genetic variants between glaucoma and anxiety, with quantitative analysis of allele frequencies and effect sizes; these genetic findings should be complemented by functional experiments to quantitatively verify the causal role of candidate variants in regulating neuroinflammatory or neuroendocrine pathways linked to both conditions. Additionally, integrating longitudinally collected quantitative data with genetic profiling in a single cohort will facilitate exploration of gene-environment interactions—such as how genetic susceptibility (quantified by polygenic risk scores) modifies the impact of chronic stress (a key environmental driver of anxiety, assessed via standardized stress scales) on glaucoma progression rates—filling critical gaps in current understanding.

## 8 Conclusion

Glaucoma and anxiety demonstrate a bidirectional association, where anxiety accelerates disease progression through neuroendocrine, vascular regulatory, and neuroinflammatory pathways, while visual function impairment and neuroinflammation induced by glaucoma exacerbate anxiety, forming a vicious cycle. Although current research has limitations in longitudinal evidence, genetic validation, and psychological intervention strategies, the multidisciplinary “mind-body integrated therapy” model has shown potential to improve patient adherence and quality of life. Future studies optimizing psychological intervention strategies guided by biomarkers and exploring the dual benefits of anti-inflammatory drugs may promote a shift in glaucoma management from mere intraocular pressure reduction to physiological-psychological collaborative intervention, bringing more comprehensive therapeutic benefits to patients.

## Data Availability

The original contributions presented in the study are included in the article/supplementary material. Further inquiries can be directed to the corresponding author.
